# Comparison of Flavor Stability of Yuja (*Citrus junos* Tanaka) Oil-Based Nano-Carriers and Dried Gels

**DOI:** 10.3390/gels11090751

**Published:** 2025-09-17

**Authors:** Seo A. Jung, Piyanan Chuesiang, Jun Tae Kim, Gye Hwa Shin

**Affiliations:** 1 Department of Food and Nutrition, Kunsan National University, Gunsan 54150, Republic of Korea; jksab@naver.com; 2 Department of Food Technology, Chulalongkorn University, Bangkok 10330, Thailand; c.piyanan@hotmail.com; 3 Department of Food and Nutrition, Kyung Hee University, Seoul 02447, Republic of Korea; 4 BioNanocomposite Research Center, Kyung Hee University, Seoul 02447, Republic of Korea

**Keywords:** Yuja, nanoemulsion, nanostructured lipid carrier, dried gels, freeze-drying, spray-drying

## Abstract

Nano-carriers and dried gels were prepared to prevent the inherent flavor of Yuja (*Citrus junos* Tanaka) from rapidly deteriorating. The properties and stability of volatile components of Yuja dried gels were compared by using colloidal systems (nanoemulsion (NE) and nanostructured lipid carrier (NLC)), coating materials (maltodextrin (MD) and a mixture of MD and β-cyclodextrin (MD/βCD)), and drying processes (spray-drying and freeze-drying). Drying was found to have a significant effect on the particle size, moisture content, color, morphology, and volatile profiles of Yuja dried gels. Meanwhile, the stability of limonene and γ-terpinene, the main volatile components of Yuja oil, was affected by the colloidal system, coating material, and storage temperature. When Yuja oil was encapsulated by an NLC and MD/βCD coating, the degradation of limonene and γ-terpinene of Yuja dried gels was reduced during storage at 4 °C.

## 1. Introduction

*Citrus junos* Tanaka (Yuja) is a well-known yellow citrus fruit in South Korea, Japan, and China. Since it contains abundant vitamin C, minerals, and citric acid, Yuja provides various human health benefits, including antioxidant, antibacterial, anti-allergy, and anti-aging properties [1,2,3,4]. In particular, the peel of Yuja contains large amounts of essential oils (EOs) that give it a fascinating and unique scent and are widely used in various processed foods such as beverages, tea, and dressings [1,5]. However, these volatile Yuja oils can be easily degraded and lost during processing and storage, altering the flavor profile of the final food product. To preserve the unique flavor or aroma of Yuja oils, it is necessary to encapsulate them in colloidal systems to protect them from harsh external environments [6].

Oil in water (o/w) nanoemulsions (NEs) and nanostructured lipid carriers (NLCs) have been reported as effective colloidal systems to encapsulate EOs and protect them from chemical degradation [7,8]. Both NEs and NLCs can encapsulate and disperse EOs as surfactant-coated tiny droplets in aqueous media [9,10]. However, NLCs differ slightly from NEs in that they have an imperfect crystalline structure due to the combination of solid lipids and liquid lipids in the system [11]. Due to the nano-scale size of the droplets, NEs and NLCs exhibit good stability and higher encapsulation efficiency compared to other systems, such as conventional emulsions and solid lipid nanoparticles [9,10,11]. Despite these advantages, NEs and NLCs have common disadvantages as they become unstable over time through the Oswald ripening process and affect particle aggregation and droplet size growth during storage [12,13]. Dried gels are prepared via the drying of gels such as aerogels, hydrogels, and cryogels, and have high porosity, a large surface area, and tunable internal structures that can improve encapsulation efficiency and protect the core materials from harsh environments [14].

Freeze-drying is a process of sublimation by freezing the moisture in the sample in the form of ice and drying it under reduced pressure [15]. Spray-drying, on the other hand, provides a dried powder product by spraying a liquid sample through an atomizer located in a hot chamber [16]. Depending on the process differences, these drying techniques have some advantages and disadvantages [16,17,18].

Apart from the drying technique, the organoleptic properties of the dried product depend on the coating materials, which provide film-forming and emulsifying properties with low viscosity at a high solid concentration to coat the core materials effectively [19]. Additionally, it is recommended that coating materials should be non-toxic, water-soluble, flavorless, and inexpensive [19]. Based on our preliminary study, maltodextrin (MD) and β-cyclodextrin (βCD) were selected for Yuja oil coating because they showed good protection of Yuja oil flavors. In addition, MD is one of the most efficient coating materials in the drying process, and βCD has a hydrophilic outer surface and a lipophilic inner structure. The mixture of MD and βCD provides an inner cavity that forms an inclusion complex, which is suitable for encapsulating lipophilic active substances such as Yuja oil [20,21].

It was hypothesized that NE and NLC systems, different coating materials (MD and MD/βCD), and drying methods would have various effects on the encapsulation and stability of Yuja oil, and that the optimal combination would enhance the preservation of the unique flavor and aroma of Yuja oil. While colloidal systems and drying processes have been commonly used to encapsulate active compounds, a comprehensive study on the effects of colloidal systems, coating materials, and drying methods on the properties and chemical stability of volatile components in Yuja oil is lacking. This study aims to fill this gap by developing Yuja dried gels using two different colloidal systems (NE and NLC) to primarily encapsulate Yuja oil into tiny droplets, as well as coating materials (MD and the mixture of MD and β-cyclodextrin (MD/βCD)) and drying processes (spray-drying and freeze-drying). Our goal is to investigate the influence of these parameters on the properties of Yuja dried gels, such as particle size, size distribution, particle morphology, moisture content, color, volatile profile, and the stability of volatile components of Yuja dried gels.

## 2. Results and Discussion

### 2.1. Particle Size and PDI of Y-NE and Y-NLC

Y-NE and Y-NLC were prepared using Yuja juice and Tween 80 (Sigma-Aldrich Co., St. Louis, MO, USA) as the continuous phase and surfactant, respectively. However, the oil phases of Y-NE and Y-NLC were different in that the former contained only Yuja oil (Korea Natural Food Co., Ltd., Jeonju, Republic of Korea), whereas the latter contained a mixture of Yuja oil, palm oil, and Tween 80. As shown in Table 1, the average particle size of Y-NE was 37.36 ± 0.43 nm and the PDI was 0.24, whereas those of Y-NLC were 32.03 ± 0.19 nm and 0.19, indicating that stable Y-NE and Y-NLC were formulated with a narrow size distribution (PDI < 0.25). However, the size and PDI of Y-NLC were significantly smaller (*p* < 0.05) than those of Y-NE, indicating that Y-NLC was more stable than Y-NE. This result is thought to be due to the partial crystalline structure of NLC resulting from the solidification of a solid lipid (palm oil) during the NLC formulation process [22].

### 2.2. Characterization of Yuja Dried Gels

#### 2.2.1. Particle Size and Size Distribution

The D_[4,3]_ and span values of Yuja dried gels obtained using different formulations and drying techniques are shown in Table 2. The spray-dried Yuja dried gels showed a smaller particle size of 11.76–12.20 μm compared to the (18.59–29.16 μm) freeze-dried samples, indicating that the drying process significantly (*p* < 0.05) affected the particle size of the dried gels. This is because the spray-drying process (Mini Spray Dryer B-290, Buchi, Essen, Germany) rapidly atomizes the compounds at high temperature through a nozzle with a constant diameter. Therefore, the particle size of the Yuja dried gels can be controlled [23]. In the freeze-drying process (Freeze Dryer, FDS8512, ilShinbiobase, Dongducheon, Republic of Korea), the dried gels appeared as a dry cake and had to be pulverized. Therefore, the type of coating material may affect the particle size of samples during pulverization. The colloidal systems (NE and NLC) and coating materials significantly (*p* < 0.05) affected the particle size of the dried gels prepared by freeze-drying. NLC-based dried gels had larger particle sizes than the NE-based dried gels, likely because NLCs contain some solid lipids (palm oil), whereas NEs contain only liquid oil. The larger particle sizes of MD/βCD in the freeze-dried samples may be due to the aggregation of β-CD [24].

The span values in this study indicate that the freeze-dried Yuja dried gels were produced with a wider size distribution than the spray-dried samples. In addition to the pulverization, these results are consistent with those of Pasrijia et al. [24], who reported a wider particle size distribution of freeze-dried microcapsules of green tea polyphenols due to the low temperature and lack of process strength required to break down the frozen liquid [24,25].

#### 2.2.2. Moisture Content and Color

Moisture content is an important parameter affecting the shelf life of spray-dried and freeze-dried products [26]. According to Klinkesorn et al. [27], a moisture content of approximately 3–4% (*w*/*w*) was reported as the highest moisture specification for dried powders in the food industry. The moisture content of the dried gels (spray-dried and freeze-dried products) was consistent with the industry specification (Table 2). However, it is important to note that the freeze-drying process produced microparticles with a significantly (*p* < 0.05) higher moisture content compared to those produced via spray-drying. This may be due to the removal of water at a lower temperature through sublimation during the freeze-drying process.

**Figure 1:**
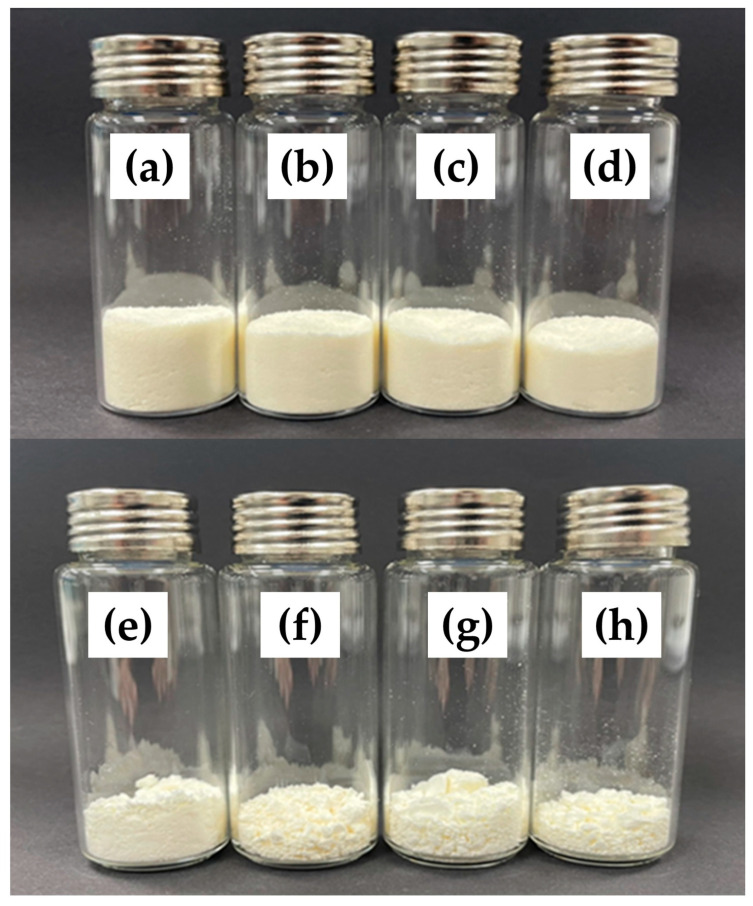
Appearance of the prepared Yuja dried gels: SDG-Y-NE/MD (**a**), SDG-Y-NE/MD/βCD (**b**), SDG-Y-NLC/MD (**c**), SDG-Y-NLC/MD/βCD (**d**), FDG-Y-NE/MD (**e**), FDG-Y-NE/MD/βCD (**f**), FDG-Y-NLC/MD (**g**), and FDG-Y-NLC/MD/βCD (**h**).

Color is one of the most important quality parameters that consumers evaluate when accepting or rejecting a food. The color parameters (L*, a*, and b* value) and the appearance of Yuja powder are shown in Table 2 and Figure 1, respectively. All spray-dried gels showed higher L* values (whiter) and a* values (less green) but lower b* values (less yellow) than the freeze-dried samples. These results indicate that the high temperature used in the spray-drying process caused more color degradation compared to the freeze-drying process (using the color parameters of Yuja juice as initial parameters). Particle size has been reported as one parameter that affects the color of microparticles, as it is related to the light scattering of samples (the smaller the particles, the larger the angle of light scattering) [28]. As reported in Section 2.2.1., the spray-dried gels were smaller in size, which means that these samples tended to appear brighter compared to the freeze-dried samples [28]. Nano-carrier systems and coating materials did not significantly (*p* > 0.05) change the moisture content and color values of Yuja dried gels.

#### 2.2.3. Field Emission Scanning Electron Microscopy (FE-SEM)

**Figure 2:**
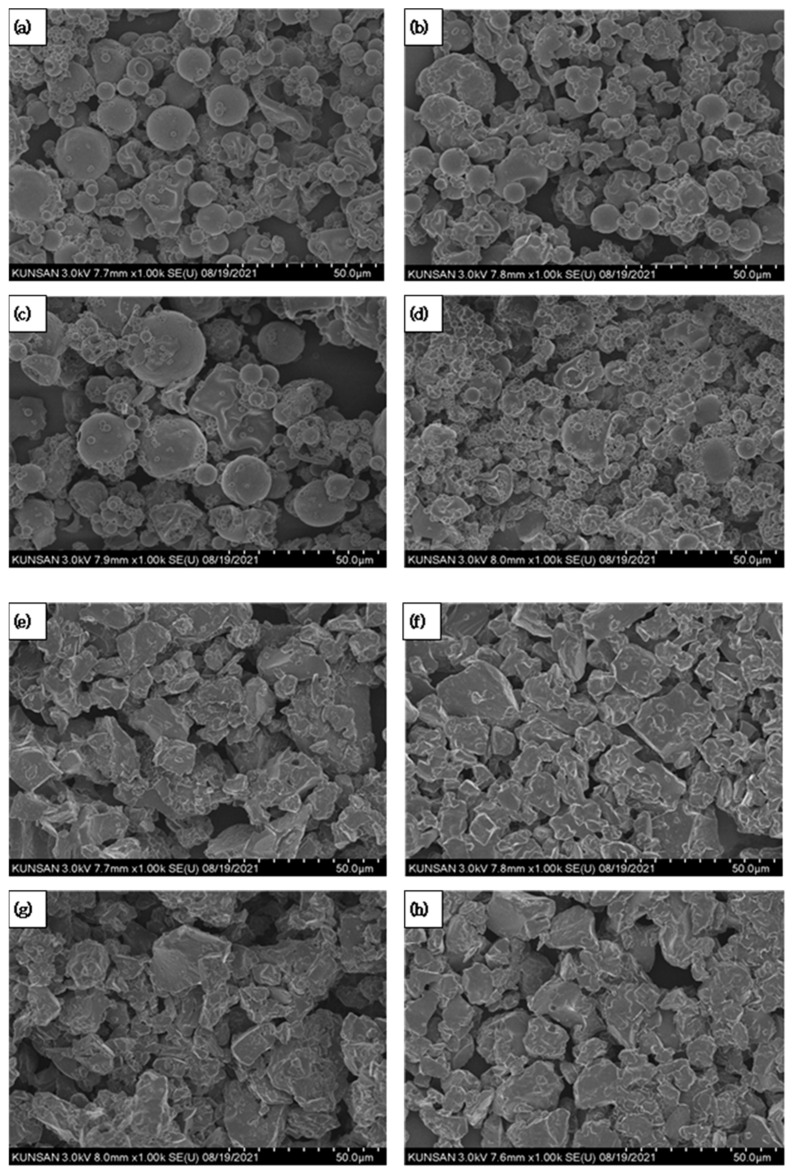
FE-SEM microphotographs of Yuja dried gels: SDG-Y-NE/MD (**a**), SDG-Y-NE/MD/βCD (**b**), SDG-Y-NLC/MD (**c**), SDG-Y-NLC/MD/βCD (**d**), FDG-Y-NE/MD (**e**), FDG-Y-NE/MD/βCD (**f**), FDG-Y-NLC/MD (**g**), and FDG-Y-NLC/MD/βCD (**h**).

The morphology of Yuja dried gels obtained by spray-drying and freeze-drying was analyzed using FE-SEM (SU 8010, Hitachi Co., Tokyo, Japan). As shown in Figure 2a–d, spray-dried gels contained spherical particles with a smooth surface, but particle deformation was observed in these samples depending on the coating materials. Compared with the other samples, the particles of SDG-Y-NLC-MD/βCD were more irregular and more agglomerate, which could be caused by (1) the non-uniform shrinkage of the droplets in the initial stage of spray-drying and (2) the self-aggregation of cyclodextrin [24]. For the freeze-dried gels, the angular and amorphous glass-like morphology observed in all samples (Figure 2e–h) could be attributed to the effect of pulverization.

#### 2.2.4. Volatile Profiles of Yuja Dried Gels Analyzed Using E-Nose-Based Fast GC

**Figure 3:**
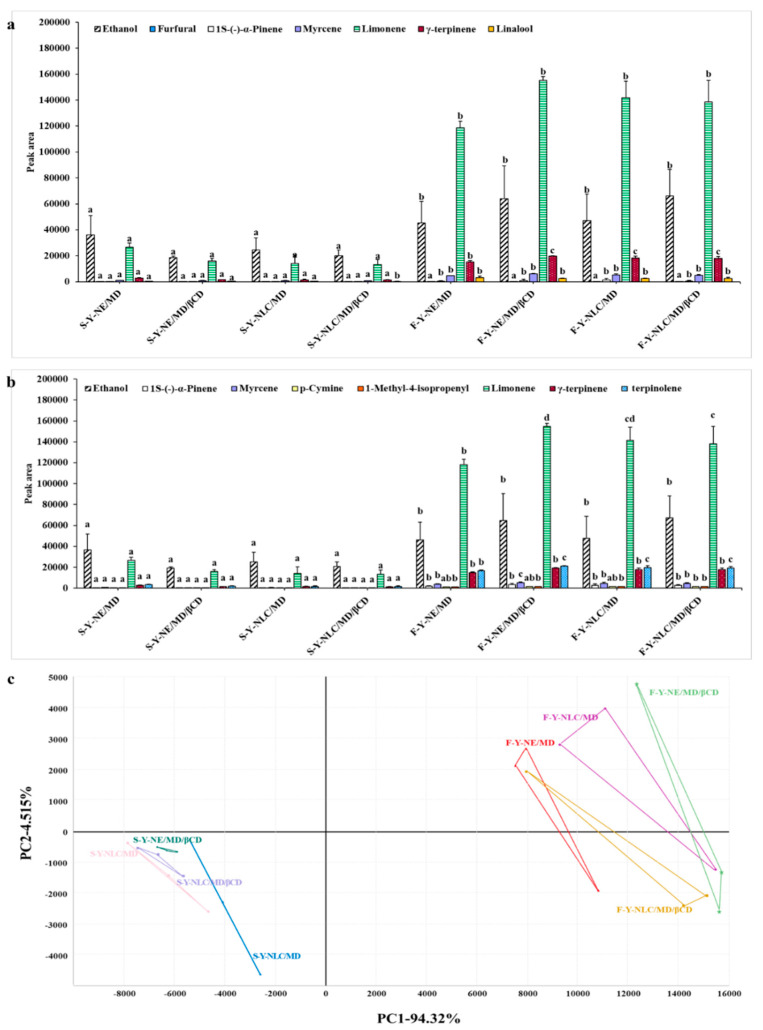
Volatile compounds contained in spray-dried and freeze-dried Yuja dried gels obtained from E-nose-based fast GC analysis: (**a**) MXT-5 column—ethanol (RT = 17.77 min), furfural (RT = 45.24 min), 1S-(-)-α-pinene (RT = 57.50 min), myrcene (RT = 60.68 min), limonene (RT = 63.64 min), γ-terpinene (RT = 66.37 min), and linalool (RT = 67.95 min); (**b**) MXT-1701 column— ethanol (RT = 19.52 min), 1S-(-)-α-pinene (RT = 54.70 min), myrcene (RT = 59.49 min), p-cymene (RT = 60.96 min), 1-methyl-4-isopropenyl1-1-cyclohexene (RT = 61.29 min), limonene (RT = 63.34 min), γ-terpinene (RT = 65.05 min), and terpinolene (RT = 66.88 min); and (**c**) PCA of data illustrating the effect of drying method on the volatile profiles of the samples. ^a–d^ Different letters in same volatile compound group indicate significant difference at *p* < 0.05 according to Duncan’s multiple range test.

The volatile profile of spray-dried and freeze-dried Yuja dried gels encapsulated with different colloidal systems and coating materials was analyzed using an E-nose-based fast GC (Heracles II, Alpha, Toulouse, France) with two separating columns of MXT-5 and MXT-1701 and an FID. Table 3 and Figure 3 show that the GC/FID of the volatile components could be distinguished in each Yuja dried gel, and 10 substances were identified, including ethanol (sweet odor), furfural (sweet, almond-like odor), 1-methyl-4-isopropenyl 1-1-cyclohexene (citrus, fruity, lemon-like odor), limonene (citrus, fruity, lemon-like- odor), γ-terpinene (citrus, fruity-like odor), linalool (floral, fruity, lemon-like odor), 1-S-(-)-α-pinene (herbaceous-like odor), myrcene (ethereal, fruity, geranium-like odor), p-cymene (citrus, fruity-like odor), and terpinolene (fruity, herbaceous, pine-like odor). The retention time (RT) of each component obtained from the MXT-5 and MXT-1701 columns is shown in Table 3 and Figure 3. Additionally, the samples obtained from the freeze-drying process were found to contain all components at peak areas approximately 5–10 times higher than those found in the spray-dried gels, indicating that the freeze-drying process is more effective than the spray-drying process in preventing the loss or degradation of volatile compounds in the samples.

PCA (Figure 3c) showed that Yuja dried gels were divided into two groups based on the overall E-nose-based fast GC data. PC1 explained 94.32% of data variability, which could distinguish spray-dried and freeze-dried gels. On the other hand, PC2 explained another 4.52% of data variability. The PCA results confirmed the effect of the drying method on the differences in the volatile profiles of Yuja dried gels, with limonene and alcohol mainly explaining the profiles of freeze-dried and spray-dried gels, respectively. To evaluate the impact of the colloidal systems and coating materials on the volatile properties of the samples, limonene and γ-terpinene (contribute to the citrus and lemon-like odor [29]) were selected as the major volatile components found in all Yuja dried gels (Figure 3a,b) and their stability during storage at different temperatures was investigated in a further experiment.

### 2.3. Storage Stability of Limonene and γ-Terpinene Contained in Yuja Dried Gels

**Figure 4:**
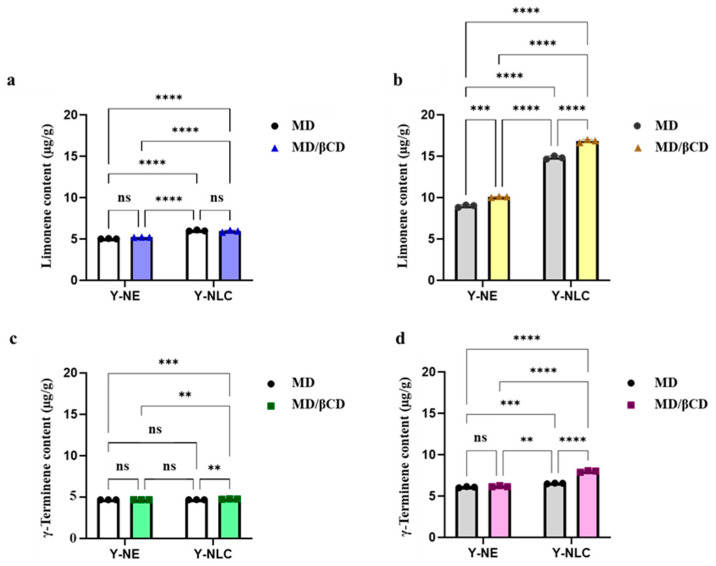
The limonene (**a,b**) and γ-terpinene content (**c,d**) contained in different Yuja dried gels; spray-dried gels (**a,c**) and freeze-dried gels (**b,d**). Asterisks on the figure indicate significant differences between each treatment, with *n* = 3; **: *p* < 0.01, ***: *p* < 0.005, and **** *p* < 0.001. ns: not significant.

To study the stability of limonene and γ-terpinene, Yuja dried gels were separately stored at 4 °C and 25 °C for 60 days. Then, the effects of colloidal systems, coating materials, and storage temperatures on the stability of those compounds were investigated at 1, 7, 14, 21, 30, and 60 days of storage. The initial contents of limonene and γ-terpinene in the samples were analyzed using GC-MS analysis immediately after drying, and the results are shown in Figure 4 (figure was prepared using GraphPad Prism 10 software). As expected, the contents of limonene and γ-terpinene in the freeze-dried gels were significantly higher (*p* < 0.05) than those of the spray-dried gels. This is because these compounds are prone to thermal degradation during the spray-drying process [30]. The colloidal systems and coating materials also affected the content of limonene and γ-terpinene in the samples. Statistical analysis revealed a significant interaction between nano-carrier systems and coating materials in determining the limonene content of Yuja dried gels formulated using spray-drying and freeze-drying techniques. For the former technique (Figure 4a,c), significant differences were observed among treatments involving different nano-carriers, suggesting a dominant effect of nano-carrier type under this drying condition. However, changing coating materials within the same nano-carrier type did not increase the content of active compounds. This result indicates a negligible effect of the coating material on the encapsulation efficiency of Yuja spray-dried gel. Under the freeze-drying condition, all Yuja dried gels showed significant differences in limonene level, which highlights the key role of nano-carrier systems and coating materials that independently and interactively influenced limonene content. The Y-NLC/MD/βCD formulation exhibited the highest limonene levels, suggesting a synergistic effect of those two factors in retaining volatile active compounds under freeze-drying conditions. The results regarding the γ-terpinene content in Yuja dried gels also show the same trend, namely that the interaction between nano-carrier systems and coating materials was more pronounced in the Y-NLC/MD/βCD formulation under freeze-drying (*p* < 0.05). According to the microstructural characteristics, the NLC contains a solid lipid matrix, providing greater stability and more effective encapsulation of lipophilic compounds compared to the NE, which consists of dispersed oil droplets in an aqueous phase [9,10,11,20,21]. Additionally, βCD is a cyclic oligosaccharide with a hydrophobic cavity. It forms inclusion complexes with high affinity for small lipophilic molecules. Therefore, βCD offers better barrier property against volatilization of compounds when compared to MD, a linear polysaccharide that provides a loosely structure protective matrix.

#### 2.3.1. Limonene Stability

**Figure 5:**
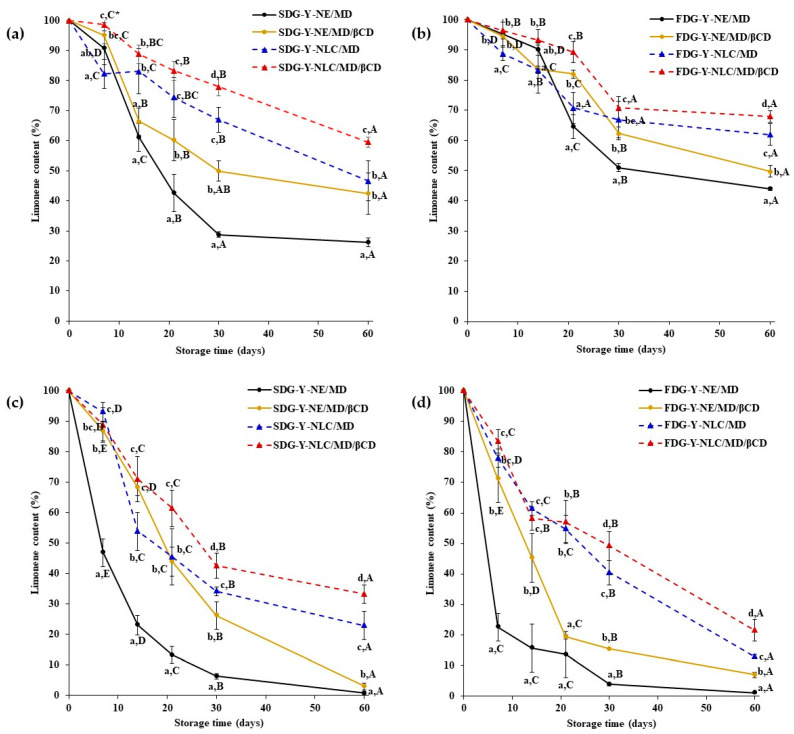
The limonene content of spray-dried gels stored at 4 °C (**a**), freeze-dried gels stored at 4 °C (**b**), spray-dried gels stored at 25 °C (**c**), and freeze-dried gels stored at 25 °C (**d**). * Different lower-case letters indicate a significant difference within the samples, and different capital letters indicate a significant difference within the storage time at *p* < 0.05.

The limonene content of each dried gel during storage is shown in Figure 5. For the spray-dried gels stored at 4 °C (Figure 5a), the limonene content decreased over the storage time of 60 days. However, the results show that the NLC combined with MD/βCD played a crucial role in effectively encapsulating the Yuja oil and protecting against limonene degradation in the dried gels. The most effective formulation in preserving limonene content was SDG-Y-NLC/MD/βCD, followed by SDG-Y-NLC/MD, SDG-Y-NE/MD/βCD, and SDG-Y-NE/MD. The NLC’s imperfect crystalline structure was pivotal in encapsulating the Yuja oil, minimizing release and structural changes of the active compound [22]. The more prominent limonene degradation-delaying ability of the NLC compared to the NE in this study is not consistent with the result previously reported by Zhang, Hayes, Chen, and Zhong [31], who explained the limited mobility of the solid lipid (anhydrous milk fat) core as the main reason for the delayed β-carotene degradation during storage. Additionally, further coating with MD/βCD significantly enhanced the encapsulating process by incorporating Yuja oil droplets into the inner cavity of βCD. This dual-layer encapsulation not only protected the active compounds from environmental degradation but also highlighted the synergistic interaction between MD and βCD, which further stabilized the core oil during storage [21,25]. This approach underscores the superior ability of the NLC and MD/βCD coatings to maintain the integrity and flavor profile of Yuja oil compared to traditional methods.

Similar results were found for the freeze-dried gels, of which FDG-Y-NLC/MD/βCD was found to be more capable of retarding limonene degradation than the other samples (Figure 5b). However, the limonene content in the freeze-dried gels stored at 4 °C was significantly higher than that in the spray-dried gels stored at the same temperature. This difference may be due to the presence of free or poorly encapsulated limonene (located at or near the surface of microparticles) in the spray-dried gels, which degraded faster than the well-encapsulated limonene in the freeze-dried gels. The rapid degradation of surface-located carotenoids in spray-dried gels has also been previously reported by Hass et al. [28].

As shown in Figure 5c,d, the degradation of limonene in spray-dried and freeze-dried gels stored at 25 °C was very similar to that in the samples stored at 4 °C, but the degradation rate was faster, and limonene content decreased sharply in all samples at the end of the storage period. These results clearly indicate that limonene degradation was significantly induced by storage temperature.

In summary, these findings highlight the synergistic effects of nano-carrier systems, coating materials, and drying methods on encapsulation efficiency for retaining active volatile compounds in Yuja oil. As previously mentioned, the NLC system used in this study promoted the encapsulation of Yuja oil into the form of fine oil droplets and prevented the oxidation and degradation of active volatile compounds due to some portion of the solid lipid. The coating material served as an additional protective barrier, improving the mechanical properties and further stabilizing the encapsulation. Finally, the drying technique converted the coated Yuja encapsulated nano-carriers into stable dried gel powders. This step impacted the particle morphology, encapsulation efficiency, and long-term stability of Yuja oil. Yang et al. [32] reported that the combination of surfactants (Tween 20 and span 20) and coating materials (whey protein isolate and maltodextrin) with spray-drying enhanced the retention of flavor compounds in raspberry.

#### 2.3.2. γ-Terpinene Stability

**Figure 6:**
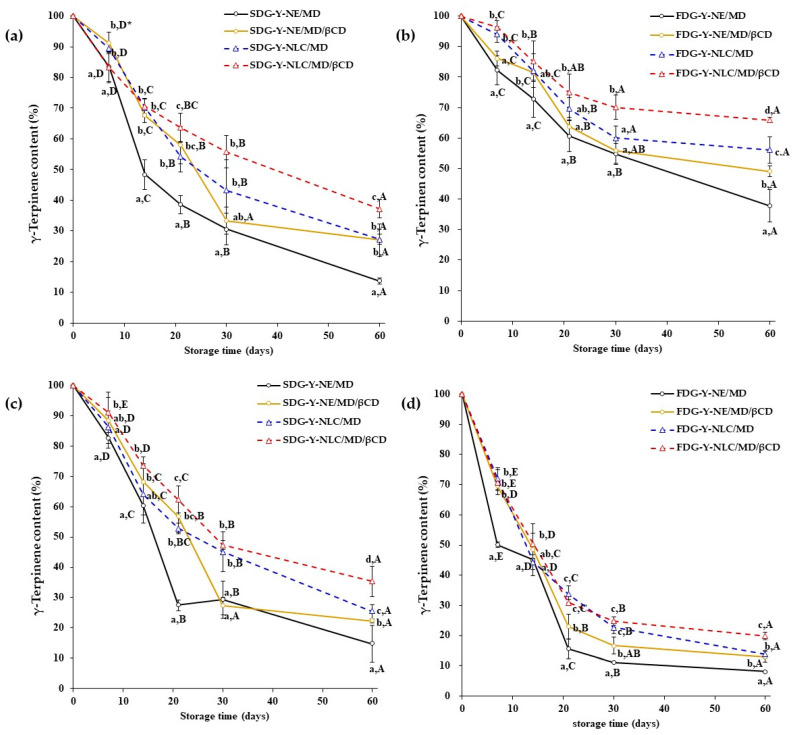
The γ-terpinene content of spray-dried gels stored at 4 °C (**a**), freeze-dried gels stored at 4 °C (**b**), spray-dried gels stored at 25 °C (**c**), and freeze-dried gels stored at 25 °C (**d**). * Different lower-case letters indicate a significant difference within the samples, and different capital letters indicate a significant difference within the storage time at *p* < 0.05.

Similar to limonene, γ-terpinene degradation was also observed during storage in each dried gel. As shown in Figure 6, the presence of Y-NLC or MD/β-CD in the formulations stored at 4 °C showed less γ-terpinene degradation than the other samples. However, the degradation of γ-terpinene seemed to be more prominent when compared to the degradation profile of limonene. For example, the γ-terpinene content of SDG-Y-NLC/MD/βCD stored at 4 °C decreased from 100% to 55.71% and 37.14% at 30 and 60 days of storage, respectively. On the other hand, the limonene contents of similar samples stored for 30 and 60 days were 83.26% and 59.53%, respectively. These results indicate that γ-terpinene has a high susceptibility to autoxidation during storage [33].

## 3. Conclusions

Yuja dried gels were successfully developed using different nano-carrier systems (NE and NLC), coating materials (MD and MD/βCD), and drying methods (spray-drying and freeze-drying). The freeze-dried Yuja gels exhibited a larger particle size, broader size distribution, and more angular shape compared to the spray-dried Yuja gels. Additionally, drying methods affected the moisture content, color, and volatile profile of the obtained particles. The freeze-dried gels contained higher characteristic volatile components of Yuja oil, including limonene and γ-terpinene, than the spray-dried gels. These results and PCA indicate that the drying techniques significantly affected the volatile profile of Yuja dried gels. The type of colloidal system and coating material also played a critical role in the stability of volatile compounds during storage. Specifically, formulations incorporating NLC and βCD demonstrated superior preservation of limonene and γ-terpinene during 60 days of storage at 4 °C and 25 °C. The reason is due to the imperfect crystalline structure of the NLC and the inner cavity in the βCD structure. These findings highlight the potential of the NLC and MD/βCD to enhance the stability and prevent the degradation of volatile components in Yuja oil. However, this study is limited by the absence of sensory evaluation in real food matrices, which is necessary to assess practical applicability. Therefore, future research will focus on applying Yuja dried gels to various food systems and evaluating their bioavailability, sensory properties, and functional performance. Drying techniques will also be adjusted to improve particle uniformity and the preservation of volatile compounds.

## 4. Materials and Methods

### 4.1. Materials

Yuja oil and Yuja juice were provided by Korea Natural Food Co., Ltd. (Jeonju, Republic of Korea). Tween 80 and palm oil were purchased from Sigma-Aldrich Co. (St. Louis, MO, USA). Maltodextrin was purchased from ES Food Co., Ltd. (Gunpo, Republic of Korea). β-cyclodextrin (purity ≥ 98%, molecular weight of 1135 Da) and maltodextrin (DE 12, food grade, 100% corn starch-based) were purchased from Daejung Chemicals & Metals Co., Ltd. (Siheung, Republic of Korea).

### 4.2. Analysis of Yuja Oil

**Figure 7:**
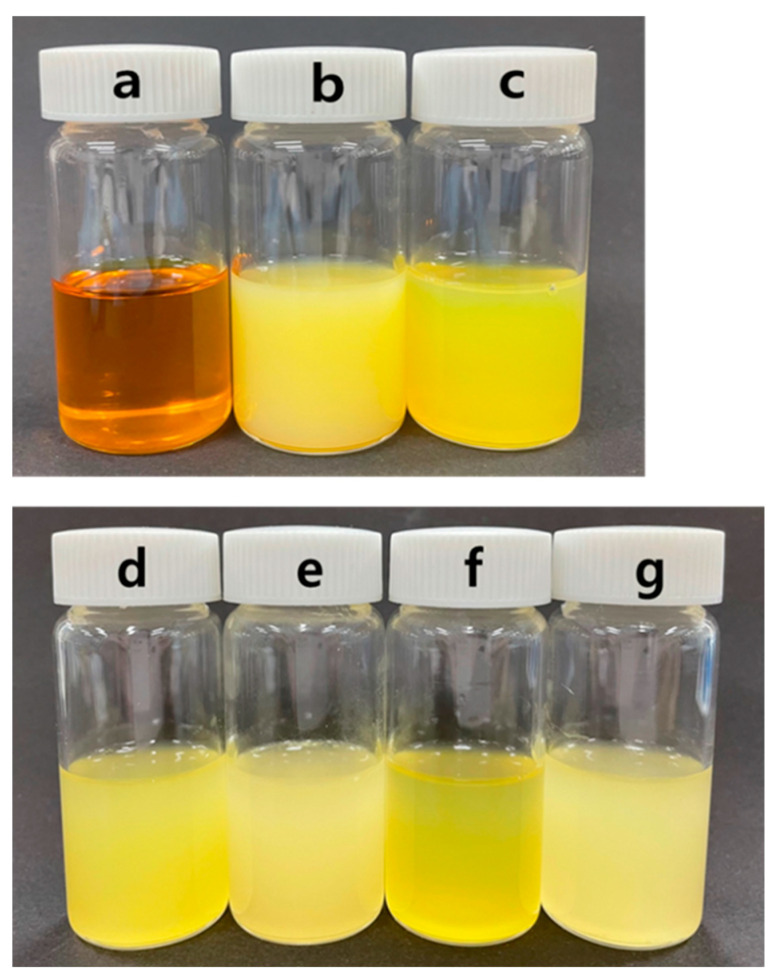
Appearance of Yuja oil (**a**), Y-NE (**b**), Y-NLC (**c**), Y-NE/MD (**d**), Y-NE/MD/βCD (**e**), Y-NLC/MD (**f**), and Y-NLC/MD/β-CD (**g**).

Yuja oil was extracted from Yuja peel using a cold-press method. The extracted Yuja oil was brown in color, as shown in Figure 7a. The volatile compounds contained in Yuja oil were analyzed using a gas chromatography–mass spectrometry (GC-MS) analysis according to Purushothaman and Ravi’s method [34]. Briefly, Yuja oil was firstly diluted 10 times in methanol before being subjected to a GC system (6890N, Agilent Technologies Inc., Santa Clara, CA, USA) equipped with a mass spectrometer system (Pegasus IV, LECO, St. Jopseph, MI, USA). The DB-5MS column (30 m × 0.25 mm × 0.25 μm, Agilent Technologies Inc.) was used as a separating column. Nitrogen gas was used as the carrier gas with a 30:1 split ratio. The injector temperature was initially kept at 30 °C for 10 min, prior to ramping it to 280 °C at 10 °C/min, then held here for 10 min. The mass selective detector was operated in electron impact ionization mode at 1750 V. GC-MS analysis showed that limonene (retention time = 1098.0 s; 12.04% total peak area) and γ-terpinene (retention time = 1127.7 s; 11.76% total peak area) were the major volatile compounds contained in Yuja oil (Figure S1 in the Supplementary Material).

### 4.3. Preparation and Characterization of O/W Yuja Oil-NE and Yuja Oil-NLC

Yuja oil-based NE (Y-NE) was prepared using an ultrasonic homogenizer. As a continuous phase, Yuja juice was filtered using a vacuum pump to remove other solids and residues, and then mixed with Tween 80 (4% *w*/*v*) as a surfactant at 25 °C for 1 h with continuous stirring. Freshly prepared Yuja oil (3.5% *w*/*v*) was dropped into a continuous phase. The O/W coarse emulsion was prepared by stirring for 2 h and homogenization at 12,000 rpm for 5 min using a high-speed homogenizer (T18 digital ULTRA-TURRAX^®^, IKA, Königswinter, Germany). To make the nano-sized emulsion, the coarse emulsion was then placed in an ice bath to avoid excessive temperature rise and subjected to an ultrasonic homogenizer (VCX-750, Sonics & Materials Inc., Sandy Hook, CT, USA) with a 13 mm diameter probe set at 750 W, 20 kHz, and 40% amplitude for 15 min [35]. The ultrasonic homogenizer probe was located ~5 mm below the mixture surface. Then, the obtained Y-NE was filtered using a 0.25 μm syringe filter.

The Yuja oil-based NLC (Y-NLC) was also prepared in the same way as the Y-NE, except for differences in the oil phase of Yuja oil (3.5% *w*/*v*) and palm oil (0.5% *w*/*v*). The oil phase was mixed at 50 °C for 1 h and then dropped into the continuous phase. After stirring for 2 h, the mixture was homogenized using a high-speed homogenizer and an ultrasonic homogenizer, and filtered in the same manner as for Y-NE preparation. Both Y-NE and Y-NLC were a yellow and cloudy solution, as shown in Figure 7b and 7c, respectively, and there seemed to be no difference to the naked eye.

The average particle size and polydispersity index (PDI) of the prepared Y-NE and Y-NLC were measured using a dynamic light scattering instrument (Zetasizer Nano-ZS90; Malvern Instruments Ltd.; Worcestershire, UK) at an angle of 173°. All measurements were performed in triplicate, and the average values were obtained.

### 4.4. Coating of Yuja Oil-Based NE and NLC

#### 4.4.1. Preparation of Y-NE and Y-NLC

To coat Y-NE and Y-NLC, the dispersions were blended with gelling materials of MD (30%, *w*/*v*) or MD/βCD (29: 1%, *w*/*v*). Each coating material was first dissolved in Yuja juice and then mixed with Y-NE or Y-NLC. The mixing ratio of the coating solution and Y-NE or Y-NLC was 1:1. The appearance of MD or MD/βCD-based Y-NE and Y-NLC is shown in Figure 7d–g.

#### 4.4.2. Spray-Dried Yuja Gels

The spray-drying process (S) of the Y-NE and Y-NLC dispersions was performed in a laboratory-scale spray-dryer (Mini Spray Dryer B-290, Buchi, Essen, Germany). The spray-drying conditions were as follows: the inlet and outlet air temperatures were 180 °C and 100 °C, respectively, and the feed flow rate was set at 4.8 mL/min. The resulting spray-dried Yuja gels were stored in a closed container at 4 °C until further analysis. Yields of Y-NE and Y-NLC dried gels obtained via the above process ranged from 27.30 to 31.22% depending on the type of coating material.

#### 4.4.3. Freeze-Dried Yuja Gels

For freeze-drying, the Y-NE and Y-NLC dispersions were frozen at −56 °C before loading into the freeze-dryer (Freeze Dryer, FDS8512, ilShinbiobase, Dongducheon, Republic of Korea). The freeze-drying process (F) was performed at −80 °C for 24 h. The obtained lyophilized samples were pulverized (350G New Type Pulverizing Machine, Rong Tsong Precision Technology Co., Taichung, Taiwan) and stored at 4 °C in a sealed container until further analysis. Yields of Y-NE and Y-NLC dried gels obtained via the freeze-drying process ranged from 88.58 to 94.51% depending on the type of coating material.

### 4.5. Characterization of Physicochemical Properties of Yuja Dried Gels

#### 4.5.1. Measurement of Particle Size and Distribution

The particle size and size distribution of the Yuja dried gels were analyzed using a laser light diffraction instrument (HELOS/RODOS & SUCELL, Sympatec GmbH, Niedersachsen, Clausthal-Zellerfeld, Germany). Particle size was expressed as the volume size D_[4,3]_ (De Brouckere average diameter), defined as the diameter of spheres of equal volume, which was calculated from triplicate measurements using Equation (1):

D_[4,3]_ = ∑N_i_D_i_^4^/∑N_i_D_i_^3^ (1)

where D_i_ and N_i_ are the particle diameter for particle fraction i and the number of particles with size D_i_, respectively. The particle size distribution of samples was reported as a span value, calculated from the characteristic parameters D10, D50, and D90 using Equation (2) [36]:

(2) $$\mathrm{Span}=\frac{D90-D10}{D50}$$

where D10, D50, and D90 are the particle size for which 10%, 50%, and 90%, respectively, of the particles are below these sizes.

#### 4.5.2. Moisture Content

The moisture content was measured using a moisture analyzer (MA100, Sartorius, Seongnam, Republic of Korea). The obtained Yuja dried gels (0.5 g) were stored at 105 °C until a constant weight was reached. Moisture content (%) was calculated with initial weight and final weight using Equation (3):

(3) $$Moisture\;content(\%)=\frac{\left(W_{initial}-W_{final}\right)}{W_{initial}}\times 100$$

#### 4.5.3. Color

Each Yuja dried gel was put into a 20 mm diameter Petri dish. The Commission Internationale de l’Eclairage (CIE) color of samples was evaluated in terms of lightness (L*), redness (a*), and yellowness (b*) on a standard white plate (L* = 96.3, a* = −0.38, b* = 1.12) using a colorimeter (Ci6X, X-Rite Inc., Grand Rapids, MI, USA).

#### 4.5.4. Field Emission Scanning Electron Microscope (FE-SEM)

The surface morphology of each Yuja dried gel was observed under a field emission scanning electron microscope (FE-SEM) (SU 8010, Hitachi Co., Tokyo, Japan). The samples were placed on carbon tape stuck with the specimen holder before gold coating [37]. The analysis was conducted at an acceleration voltage of 3 kV.

#### 4.5.5. Electronic Nose-Based Fast GC Analysis

Volatile components in the headspace of Yuja dried gels (1 g) were analyzed using an electronic nose (E-nose)-based fast GC system (Heracles II, Alpha, Toulouse, France) equipped with two parallel connected columns (MXT-5, a non-polar column (10 m × 0.18 mm × 0.4 μm film thickness), and MXT-1701, a mid-polar column (10 m × 0.18 mm × 0.4 μm film thickness)) and coupled with a flame ionization detector (FID) [38]. The analytes were separated using the following temperature program: the initial temperature of 50 °C was ramped at a rate of 1 °C/s to 80 °C and 3 °C/s to 250 °C (kept for 2 min). The carrier gas flow rate was 1 mL/min. The flavor components of each sample were calculated from the relative retention index of Kovats. Data analysis was then performed using the AroChemBase V6 (AlphaSoft version 14.2) to compare those Kovats indexes. The relative retention index of Kovats is a basis identification factor of the AroChemBase software described as an index that compares retention time between molecules and normal alkanes under similar conditions [39].

#### 4.5.6. Stability of Volatile Compounds Contained in Yuja Dried Gels During Storage

Two compounds, limonene, and γ-terpinene, which were observed as the major constituents of Yuja oil (Section 2.1), were selected and their stability was investigated during dark storage at 4 °C and 25 °C for 60 days. Limonene and γ-terpinene were collected from the headspace of the samples using a headspace sampler (7679A, Agilent Technologies, Santa Clara, CA, USA) at 60 °C for 15 min and then applied to a GC system equipped with an FID (7890B, Agilent Technologies). A DB-5MS ultra-inert column (30 m × 0.25 mm × 0.25 μm, Agilent Technologies) was used as a separating column, and nitrogen gas was used as the carrier gas with a split ratio of 30:1. The injector temperature was 280 °C according to the method of Pelicer et al. [25]. The initial temperature was maintained at 60 °C, then increased to 160 °C at a rate of 4 °C/min, 232 °C at a rate of 6 °C/min, and 248 °C at a rate of 8 °C/min. The temperature was then held at 248 °C for 5 min. The total running time was 45 min. The limonene and γ-terpinene concentrations of each sample were analyzed on 1st, 7th, 30th, and 60th days of storage.

### 4.6. Statistical Analysis

The results were reported as the mean ± standard deviation from triplicate experiments. The outcomes were subjected to one-way analysis of variance (ANOVA) using SPSS software version 24.0 (SPSS Inc., Chicago, IL, USA). Significant differences between means were compared using Duncan’s multiple range test at a significance level of *p* < 0.05. Principal components analysis (PCA), an unsupervised multivariate method, was used to evaluate the differences in volatile profile data of various formulations of spray-dried and freeze-dried Yuja powders obtained from the E-nose-based fast GC analysis. Once the data matrix was submitted to PCA (AlphaSoft version 4.2, Alpha Mos, Toulouse, France), the PC scores were calculated, and the dimensionality of the data was visualized on the PC score plot (PC1 versus PC2).

## Figures and Tables

**Table 1:** Average particle size and polydispersity index (PDI) of Yuja NE and Yuja NLC.

Formulations	Average Particle Size (nm)	PDI
Y-NE	37.36 ± 0.43 ^b^	0.24 ± 0.00 ^b^
Y-NLC	32.03 ± 0.46 ^a^	0.19 ± 0.00 ^a^

^a,b^ Different letters in the same column indicate a significant difference at *p* < 0.05 (*n* = 3).

**Table 2:** Particle size, span values, moisture content, and color parameters of various Yuja dried gels.

Samples	D_[4,3]_ (μm)	Span	Moisture Content (%)	Color Values			
				L*	a*	b*	∆E
SDG-Y-NE/MD *	11.76 ± 0.40 ^a^	1.69 ± 0.07 ^b^	2.80 ± 0.54 ^ab^	94.32 ± 0.07 ^de^	−0.56 ± 0.06 ^e^	9.48 ± 0.28 ^b^	9.48 ± 0.28 ^b^
SDG-Y-NE/MD/βCD	11.84 ± 0.32 ^a^	1.49 ± 0.07 ^a^	2.72 ± 0.45 ^ab^	94.21 ± 0.41 ^d^	−0.80 ± 0.03 ^d^	8.21 ± 0.04 ^a^	8.21 ± 0.04 ^a^
SDG-Y-NLC/MD	12.11 ± 0.15 ^a^	1.86 ± 0.03 ^c^	2.33 ± 0.46 ^a^	94.36 ± 0.17 ^de^	−0.44 ± 0.06 ^f^	10.72 ± 0.10 ^d^	10.72 ± 0.10 ^d^
SDG-Y-NLC/MD/βCD	12.20 ± 0.18 ^a^	1.70 ± 0.08 ^b^	2.32 ± 0.23 ^a^	94.76 ± 0.34 ^e^	−0.85 ± 0.02 ^d^	9.82 ± 0.12 ^c^	9.82 ± 0.12 ^c^
FDG-Y-NE/MD	18.59 ± 0.35 ^b^	2.49 ± 0.06 ^e^	3.31 ± 0.21 ^b^	92.15 ± 0.36 ^b^	−1.66 ± 0.01 ^c^	14.29 ± 0.11 ^e^	14.29 ± 0.11 ^e^
FDG-Y-NE/MD/βCD	24.63 ± 0.11 ^c^	2.17 ± 0.02 ^d^	3.27 ± 0.11 ^b^	92.53 ± 0.25 ^b^	−1.77 ± 0.03 ^b^	14.22 ± 0.21 ^e^	14.22 ± 0.21 ^e^
FDG-Y-NLC/MD	28.52 ± 0.22 ^d^	2.23 ± 0.02 ^d^	3.17 ± 0.33 ^b^	93.02 ± 0.01 ^c^	−1.73 ± 0.02 ^b^	14.57 ± 0.13 ^f^	14.57 ± 0.13 ^f^
FDG-Y-NLC/MD/βCD	29.16 ± 0.40 ^e^	2.22 ± 0.00 ^d^	3.06 ± 0.10 ^b^	93.27 ± 0.27 ^c^	−1.86 ± 0.03 ^a^	14.16 ± 0.11 ^e^	14.16 ± 0.11 ^e^

^a–e^ Different letters in the same column indicate a significant difference at *p* < 0.05 according to Duncan’s multiple range test. * SDG: spray-dried gels; F: freeze-dried gels; Y: Yuja; NE: nanoemulsion; NLC: nanostructured lipid carrier; MD: maltodextrin. βCD: β-Cyclodextrin.

**Table 3:** Value of area of volatile compounds detected in Yuja dried gels by E-nose with Kovats index.

Column	Drying Type	RT	Samples				Compound	Description
			Y-NE/MD	Y-NE/MD/β-CD	Y-NLC/MD	Y-NLC/MD/β-CD		
MTX-5	SD	63.64	26,605 ^a^ ± 3901	15,987 ^a^ ± 2382	14,181 ^a^ ± 7895	13,273 ^a^ ± 4839	Limonene	Citrus, Fruity, Lemon
	FD		118,644 ^b^ ± 6199	155,237 ^c^ ± 3572	141,951 ^c^ ± 15,350	138,551 ^c^ ± 20,194		
	SD	66.37	2874 ^a^ ± 432	1573 ^a^ ± 245	1380 ^a^ ± 829	1259 ^a^ ± 439	γ-Terpinene	Citrus, Fruity
	FD		15,349 ^b^ ± 977	19,751 ^c^ ± 568	18,383 ^c^ ± 1872	18,032 ^c^ ± 1932		
	SD	67.95	600 ^a^ ± 52	375 ^a^ ± 44	359 ^a^ ± 161	308 ^a^ ± 90	Linalool	Floral, Fruity, Lemon
	FD		3231 ^b^ ± 1304	2703 ^b^ ± 171	2567 ^b^ ± 319	2697 ^b^ ± 1046		
MTX-1701	SD	59.49	859 ^a^ ± 123	527 ^a^ ± 98	475 ^a^ ± 264	471 ^a^ ± 161	Myrcene	Ethereal, Fruity, Geranium
	FD	59.49	3993 ^b^ ± 129	5344 ^c^ ± 680	4722 ^b^ ± 1053	4729 ^b^ ± 582		
	SD	60.96	301 ^a^ ± 94	213 ^a^ ± 86	220 ^a^ ± 125	209 ^a^ ± 105	*p*-Cymene	Citrus, Fruity
	FD	60.96	1288 ^ab^ ± 19	1597 ^ab^ ± 125	1557 ^ab^ ± 181	1499 ^b^ ± 117		
	SD	63.34	17,362 ^a^ ± 12537	15,957 ^a^ ± 2440	14,238 ^a^ ± 7755	13,416 ^a^ ± 4970	Limonene	Citrus, Fruity, Orange
	FD	63.34	118,128 ^b^ ± 6259	154,519 ^d^ ± 3632	141,382 ^cd^ ± 15,306	137,940 ^c^ ± 20,286		
	SD	66.28	3283 ^a^ ± 469	1906 ^a^ ± 337	1737 ^a^ ± 854	1618 ^a^ ± 714	Terpinolene	Fruity, Herbaceous, Pine
	FD	66.28	16,558 ^b^ ± 1245	21,163 ^c^ ± 558	19,721 ^c^ ± 1999	19,244 ^c^ ± 2038		

SD: spray-drying; FD: freeze-drying. Data values are expressed mean ± SD (*n* = 3). ^a–d^ Different letters in same group indicate significant difference at *p* < 0.05 according to Duncan’s multiple range test.

## Data Availability

The data presented in this study are available on request from the corresponding author. The data are not publicly available due to ethical reason.

## Supplementary Materials

The following supporting information can be downloaded at: https://www.mdpi.com/article/10.3390/gels11090751/s1, Figure S1. GC-MS analysis of volatile compounds in Yuja oil: (a) Limonene and (b) γ-terpinene.
