# Supplementary figures and images for: Comparison of Flavor Stability of Yuja (Citrus junos Tanaka) Oil-Based Nano-Carriers and Dried Gels
Source: Gels. 2025 Sep 17;11(9):751. doi: 10.3390/gels11090751 (PMC12469294; doi:10.3390/gels11090751)

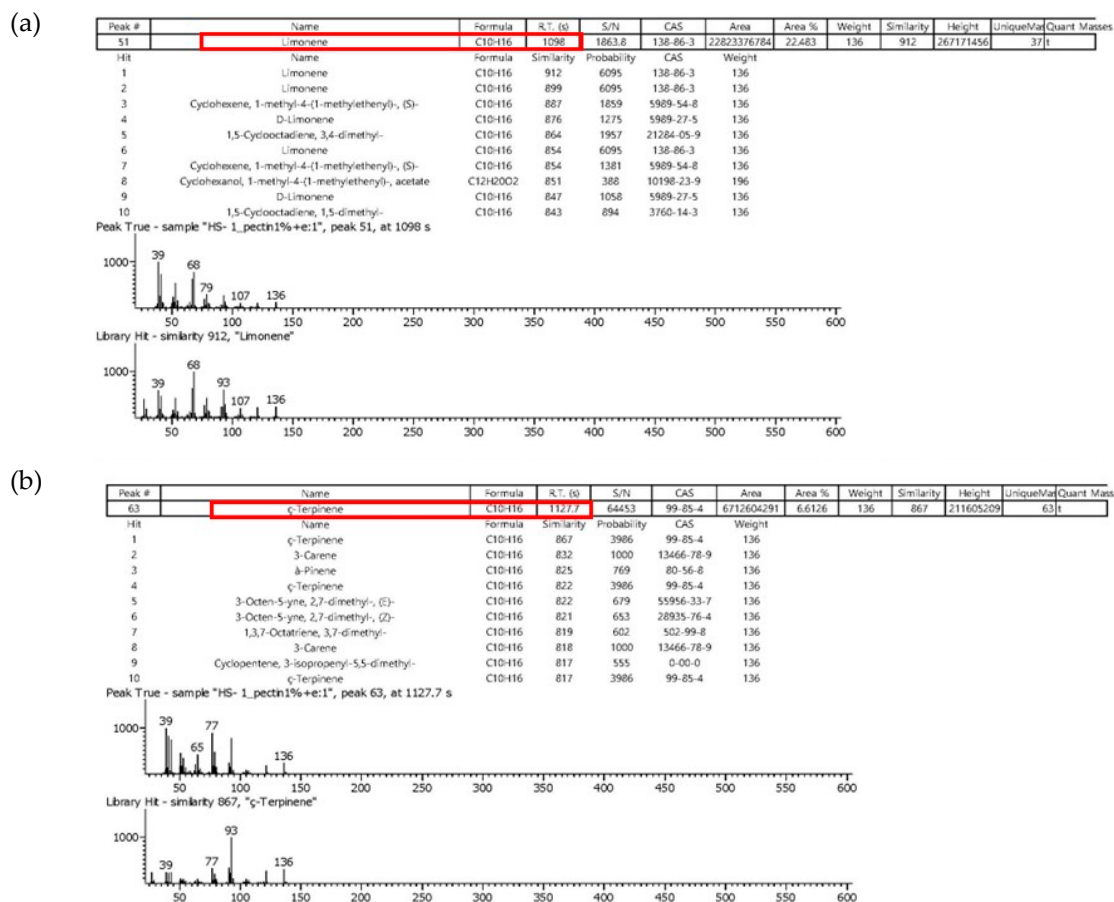

Figure S1. GC-MS analysis of volatile compounds in Yuja oil: (a) Limonene and (b)  $\gamma$ -terpinene.

Supplement: Supplementary file 1 [file gels-11-00751-s001.zip › gels-3833562-supplementary.pdf]
